# A Novel Nonsense Mutation in *FERMT3* Causes LAD-III in a Pakistani Family

**DOI:** 10.3389/fgene.2019.00360

**Published:** 2019-04-24

**Authors:** Saba Shahid, Samreen Zaidi, Shariq Ahmed, Saima Siddiqui, Aiysha Abid, Shabbir Malik, Tahir Shamsi

**Affiliations:** ^1^Department of Genomics and Clinical Genetics, National Institute of Blood Diseases and Bone Marrow Transplantation, Karachi, Pakistan; ^2^Department of Pediatrics, National Institute of Blood Diseases and Bone Marrow Transplantation, Karachi, Pakistan; ^3^Center of Human Genetics and Molecular Medicine, Sindh Institute of Urology and Transplantation, Karachi, Pakistan; ^4^Department of Clinical Hematology, National Institute of Blood Diseases and Bone Marrow Transplantation, Karachi, Pakistan

**Keywords:** primary immunodeficiency, leukocyte adhesion deficiency type III, targeted next generation sequencing, *FERMT3* gene, mutation screening

## Abstract

Leukocyte adhesion deficiency-III (LAD3) is an extremely rare primary immunodeficiency disorder, transmitted with autosomal-recessive inheritance. It is caused by genetic alteration in the *FERMT3* gene, which leads to abnormal expression of kindlin-3. This cytoplasmic protein is highly expressed in leukocytes and platelets, and acts as an important regulator of integrin activation. LAD3 has features like bleeding syndrome of Glanzmann-type and leukocyte adhesion deficiency. *FERMT3* mutation(s) have not been well characterized in Pakistani patients with LAD3. In this study, an infant and his family of Pakistani origin, presenting with clinical features of LAD, were investigated to determine the underlying genetic defect. Targeted next generation sequencing (TGS) and Sanger sequencing were performed to identify and confirm the causative mutations, respectively, and their segregation within the family. A novel, homozygous *FERMT3* nonsense mutation (c.286C > T, p.Q96^∗^) was found in the proband, and its co-segregation with LAD3 phenotype within the family was consistent with an autosomal recessive inheritance. Both parents were carriers of the same mutation. This family was offered prenatal diagnosis during first trimester of the subsequent pregnancy; the fetus carried the variant. In conclusion, our study is the first report to identify the novel homozygous variant c.286C > T, p.Q96^∗^in the *FERMT3* gene, which might be the causative mutation for LAD3 patients of Pakistani origin.

## Introduction

Leukocyte adhesion deficiency (LAD) is a primary immunodeficiency disorder caused by a defect in neutrophil adhesion to the vessel endothelium. There are three different types of this disease, and LAD3, also known as LAD1 variant (LAD1V), is the most rare form (AlmarzaNovoa et al., 2008). Additional manifestations of this disease include bleeding diathesis similar to what occurs in the Glanzmann thrombasthenia, which can, however, be excluded by normal platelet aggregation tests (Boudreaux et al., 2010). LAD3 is caused by a genetic defect in the *FERMT3* gene. This defect leads to abnormal expression of kindlin-3, a protein whose major role is the regulation of integrin activation, which is essential for the adhesion of leukocytes and platelets (Robert et al., 2011).

Genetic mutations in the *FERMT3* gene (OMIM 607901) run in autosomal recessive pattern in LAD-3 (OMIM 612840) families. *FERMT3* also known, as *KIND3*, *MIG2B*, *UNC112C*, *URP2*, or *URO2SF*, is located on chromosome 11q13.1. It encodes kindlin-3, a cytoskeleton protein involved in the stabilization and activation of the glycoprotein receptor integrin through attachment to its beta subunit. These interactions are responsible for maintaining a stable integrin conformation and to activate its subunits (Ley et al., 2007). Genetic alterations in the *FERMT3* gene cause disruption of the adherent property of integrin on both leukocytes and platelets, possibly due to defect in integrin; its structure is intact, but activation (and thus binding) is not appropriate (Svensson et al., 2009; Zimmerman, 2009).

LAD3 and LAD1 have similar clinical manifestations i.e., leukocytosis, delay in the detachment of the umbilical cord, and critical life-threatening bacterial infections. In addition, there is platelet aggregation dysfunction, which results in severe bleeding episodes. This disorder has mostly been reported in patients of Turkish, Arab Maltese or African American origin. In the present study, we used targeted next-generation sequencing (TGS) technology, the advance methodology (Zhu et al., 2017), and found a novel homozygous mutation in the *FERMT3* gene in a Pakistani family with autosomal recessive LAD3. Sanger sequencing-based prenatal diagnosis was offered to the family for the successive pregnancy, and it confirmed the co-segregation of this genetic mutation with the phenotype in this family.

## Case Presentation

### Clinical Report

The index patient is a seven-month-old boy born to first cousins parents, presenting with a prolonged history of fever and recurrent infections for 4 months. Parents reported intermittent bleeding episodes from the nose, mouth, and anus that, during patient hospitalization, were unsuccessfully treated with broad-spectrum antibiotics and packed red cells and platelets transfusion. Examination revealed a failure to thrive in the child, with both height and body weight below the 3rd percentile. He had severe pallor, bruises all over the body, and there were bilateral anterior and posterior cervical palpable lymph nodes, which were firm and tender. The liver was also palpable; it was 9 cm in span, soft and non-tender, while a firm spleen was also palpable 3 cm in its longitudinal axis. The previous record had shown bicytopenia and leukocytosis, growth of multiple microorganisms in blood, including *Burkholderia cepacia* and *Staphylococcus aureus*, and persistently high inflammatory markers. Extensive investigations done during this admission confirmed the anemia, thrombocytopenia, and leukocytosis. Bone marrow aspiration and trephine biopsy showed cellular marrow. Basic primary immunodeficiency workup showed normal immunoglobulin, while flow cytometry revealed normal CD18 expression. There was strong suspicion of primary immunodeficiency due to the persistent leukocytosis and recurrent infections.

## Methods

### Ethics Statement, Consent Statement, and Proband

**FIGURE 1:**
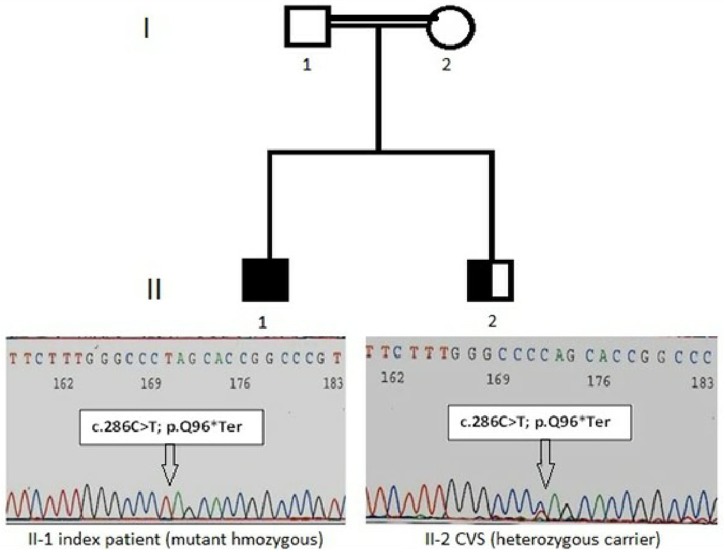
Pedigree with LAD-3 diseases in the index patient. The patient (II-l) indicates the homozygous nonsense variant of the FERMT3 gene in an electropherogram: NM_178443:c.286C > T and CVS (II-2) indicate with nonsense mutation as heterozygous carrier.

The study protocol was in accordance with the Institutional Review Board (ERC/IRB) and conformed to the tenets of the Declaration of Helsinki. Written informed consent was obtained from the parent of the patient for the publication of this case report. This study consisted of the proband and three closely related family members from two generations, with history of consanguineous marriage, described by their genetic workup and pedigree analysis in Figure 1.

### Targeted Next Generation Sequencing

Peripheral blood samples of the family were drawn and DNA was extracted using a QIAamp DNA Blood Mini Kit (Qiagen), following manufacturer’s instructions. The targeted next generation sequencing was performed using the Illumina TruSight^TM^. Inherited Disease sequencing panel, a disease targeted sequencing research panel focusing on 552 genes in regions known to harbor recessive pediatric pathogenic mutations. This panel targets 2.25 Mb of the human genomic content, with fragments of ∼500 bp. The medium coverage of the sample was >95% of amplicons at >100× coverage. Library was constructed by capturing targeted region using TruSight^TM^ rapid capture. Enriched libraries were loaded onto flow cell (Illumina, CA, United States) and paired-end sequencing runs were processed on a MiSeq (Illumina^TM^) genome sequencer. Data analysis alignment was performed with on-instrument MiSeq reporter software. The mutations identified as pathogenic were confirmed using Sanger method according to the standard protocol (BigDye^^®^^ Terminator v3.1 Cycle Sequencing Kit, Applied Biosystems^^®^^).

### Sanger Sequencing

Polymerase chain reaction (PCR) amplification and Sanger sequencing of the TGS-identified variant was performed to confirm TGS results. Primers listed in Table 1 surrounding the identified mutation were used to amplify a product of 332 bp, which was then sequenced by Sanger sequencing on an ABI-3500 sequencer instrument (Applied Biosystems Inc., Foster City, CA, United States).

**Table 1:** Primer sequences.

Primer	Sequence 5′-3′	Product size	TM
Forward (FERMT3-F)	CTGAATCCTGGGGTTGTGCT	332 bp	62°C
Reverse (FERMT3-R)	GAATCAGCCGGGCAACTTAC		


## Results

### Mutation Screening by Targeted Next Generation Sequencing

The identification of the severe immunodeficiency-causing gene mutations, through targeted inherited diseases sequencing panel, was performed on the index patient gDNA sample. Disease causing mutations were identified by the VariantStudio software and Variant Interpreter tool (Illumina). These interpretation modules can call variants automatically; options are available to apply stringent filters and for the annotation of NGS data (Hu et al., 2017). The *in silico* prediction tools SIFT, Polyphen 2, MutationTaster, MutationAssessor, dbSNP, and COSMIC were applied to filter pathogenic, benign and variant of uncertain significance. Some of the newly identified variants were not present in any of the above-mentioned public databases, and would need further verification. Interestingly, a single homozygous nucleotide substitution (c.C286T) in the exon 3 of the *FERMT3* gene (NM_178443) was identified in this index patient. This mutation leads to an amino acidic change from glutamine (Gln, Q) to a stop codon in position 96 of the kindlin-3 protein (p.Q96^∗^). Additional details of the identified sequence variant c.C286T (p.Q96^∗^), together with associated pathogenic effects, are mentioned in Table 2 and Figure 1. This variant was seemed to be novel, as it could not be identified by searching in other databases like dbSNP, COSMIC, and HGMD (Table 2). Variants identified in other genes for *EVC, DPYD, COL4A3* and *TSPYL1* by NGS were excluded as not having a damaging effect assessed by prediction tools on proteins. Finally, this family was offered prenatal diagnosis during subsequent pregnancy, which was performed by chorionic villous sampling done during the 11th week of gestation and Sanger sequencing; the fetus was found to be heterozygote for the same mutation c.C286T (p.Q96^∗^) (Figure 1).

**Table 2:** *FERMT3* variant identified in a LAD3 patient.

		Variation				
Gene	Exon	Nucleotide	Protein	Type	Status	dbSNP
*FERMT3*	03	c.C286T	p.Q96^∗^	Homozygous	Nonsense	Novel


*FERMT3, leukocyte adhesion deficiency-III; c, variation at cDNA level; p, variation at protein level; ^∗^stop codon*.

## Discussion

Leukocyte adhesion deficiency-III (LAD3) is a rare and recently identified primary immunodeficiency, which has different genetic mutations than the ones present in the other two LAD types. In the current study, we found a novel homozygous, stop codon variant c.C286T (p.Q96^∗^) in the *FERMT3* gene in a Pakistani family. The protein structure of *FERMT3* comprises of a FO domain, an F1 domain, an F2 with PH domain and an F3 domain that have the binding site to integrin beta subunit. The identified mutation lies within the FO domain in exon 3, and is different from the mutations that were previously identified at the N-terminal of the protein, specifically in the pleckstrin homology and FERMT3 sub domains (Robert et al., 2011). Robert et al. (2011) reported a p.N54Rfs142 mutation at a splice site within the FO domain. In that report, *in vitro* studies suggested that this mutation was causing a decrease in the mRNA level resulting in an unstable transcript. The nonsense mutation p.Q96X that we identified in this study is also lying within the FO domain. Similarly, nonsense mutations leading to defects in protein expression were reported in patients of Turkish (Mory et al., 2008; Kuijpers et al., 2009; Svensson et al., 2009), Arab (Kuijpers et al., 2009; Malinin et al., 2009), Maltese (Svensson et al., 2009), and African American origin (McDowall et al., 2010).

To date, very few cases have been described for Leukocyte Adhesion Deficiency all over the world; most of the affected individuals (323) were diagnosed with LAD1 (AlmarzaNovoa et al., 2008),while LAD-3 seems to be more sporadic. It is possible that LAD is reported with even lower frequency, due to the failure in correctly diagnosing rare entities. LAD3 cases caused by genetic mutations in *FERMT3* were reported in Turkish and Maltese patients; a homozygous nonsense mutation (R509X) was reported in the Turkish patients, while the Maltese patient was found to be homozygous for an A-to-G substitution in exon 14 at the splice acceptor site (Svensson et al., 2009). Expression studies confirmed that both mutations were destabilizing KINDLIN3 mRNA. However, in these studies, Western blotting showed no expression of KINDLIN3 protein in the patients, whereas expression was normal in their parents.

A novel p. R573X nonsense mutation in *FERMT3* was reported in a Turkish patient, while p.W229X in Arabic patients. *In vitro* studies revealed that *FERMT3* protein was not present in leukocytes and platelets of all tested patients, which had, however, similar defects in neutrophil and platelet function (Kuijpers et al., 2009). In addition to its adhesion properties, *FERMT3* gene product is also involved in leukocyte migration. This was confirmed by the *in vitro* effects of the homozygous mutations (G308R and 1275delT) in the *FERMT3* gene, which were the cause of severe LAD3 in an African American girl (McDowall et al., 2010). Almost all cases of LAD III were diagnosed with innate immune defects. However, Suratannon et al. (2016) identified p.Gln599Ser mutation in *FERMT3* gene in Thai patient that presented with humoral immune defect (Suratannon et al., 2016). Table 3 summarizes all the mutations in the *FERMT3* identified in the literature. To the best of our knowledge, this *FERMT3* variant is a novel mutation that broadens the mutation spectrums of LAD3. Thus, this finding shows that the recessive *FERMT3* mutation c.C286T (p.Q96^∗^) likely caused LAD-3 in our studied Pakistani pedigree.

**Table 3:** List of reported *FERMT3* mutations in LAD3.

S. No	Nucleotide change	Protein change	Type	References
1	c.286C > T	p.Gln96X	Nonsense	Current study
2	c.1069C > T	p. Arg357X	Nonsense	Wolach et al., 2019
3	c.1795C > T	p.Gln599Ser	Missense	Suratannon et al., 2016
4	c.1597C > T	p.Gln533X	Nonsense	Crazzolara et al., 2015
5	c.1426C > T	p.Gln476X	Nonsense	Harris et al., 2012
6	c.238_244dup	p.Lys82ThrfsX67	Insertion	Van de Vijver et al., 2012
7	c.922G > A	p. Gly308Arg	Missense	McDowall et al., 2010
8	c.1275delT	p.Glu426ArgfsX3	Frame shift	McDowall et al., 2010
9	c.161-2A > C	p.Asn54ArgfsX142	Splice site	Robert et al., 2011
10	c.687G > A	p.Trp229X	Nonsense	Manevich-Mendelson et al., 2009; Robert et al., 2011
11	c.48G > A	p.Trp16X	Nonsense	Malinin et al., 2009
12	c.1671-2A > G	Deletion exon 14 p.Phe558TrpfsX141	Splice site	Svensson et al., 2009
13	c.1729C > T	p.Arg577X	Nonsense	Kuijpers et al., 2009
14	c.1717C > T	p.Arg573X	Nonsense	Kuijpers et al., 2009; Jurk et al., 2010
15	c.1525C > T	p.Arg509X	Nonsense	Mory et al., 2008; Kuijpers et al., 2009; Malinin et al., 2009; Svensson et al., 2009
16	c.1537C > T	p.Arg513X	Nonsense	Mory et al., 2008; Kuijpers et al., 2009; Malinin et al., 2009; Svensson et al., 2009


*FERMT3, leukocyte adhesion deficiency-III; c, variation at cDNA level; p, variation at protein level*.

## Conclusion

In conclusion, this study wants to stress the importance of early diagnosis. As in the majority of primary immunodeficiency diseases, the prognosis of LAD3 is extremely dependent on early age diagnosis, with timely management of bacterial infections and consideration for HSCT. In addition, this autosomal recessive disorder has high incidence in areas with high rate of consanguineous marriages. Therefore, broadening the spectrum of known mutations underlying the phenotype of such a life-threatening disease can help offering and performing better genetic counseling and prenatal diagnosis.

## Ethics Statement

This study was carried out in accordance with the recommendations Institutional Review Board (ERC/IRB). The protocol was approved by the Institutional Review Board (ERC/IRB). Written informed consent was obtained from the parents of the subjects in accordance with the Declaration of Helsinki.

## Author Contributions

SabS contributed to the study design, data interpretation, and manuscript writing. SZ and SaiS were responsible for clinical examination and evaluation of patient and family. SA performed the laboratory work. AA contributed to the bioinformatics analysis. SM and TS were involved in study design, patients’ recruitment, and supervised the study and reviewed the manuscript.

## Conflict of Interest Statement

The authors declare that the research was conducted in the absence of any commercial or financial relationships that could be construed as a potential conflict of interest.
